# Description of New Morphological Variation of *Culex* (*Culex*) *coronator* Dyar and Knab, 1906 and First Report of *Culex* (*Carrollia*) *bonnei* Dyar, 1921 Found in the Central Region of Peru

**DOI:** 10.1007/s13744-024-01160-7

**Published:** 2024-06-25

**Authors:** Eddyson Montalvo-Sabino, Omayra P. Marquez-Ocaña, Griselda A. Otiniano-Moreno, Miguel A. Chuquiyauri-Talenas, Tiago Melo, Gonçalo Seixas, Ricardo Parreira, António Paulo Gouveia de Almeida

**Affiliations:** 1 Hermilio Valdizan National University (UNHEVAL), Huanuco, Peru; 2 Global Health and Tropical Medicine, GHTM, Associate Lab in Translation and Innovation Towards Global Health, LA-REAL, Institute of Hygiene and Tropical Medicine (IHMT), Univ Nova de Lisboa (NOVA), Lisboa, Portugal

**Keywords:** *CoxI*, Barcode, Mosquitoes, Culicidae, Genitalia

## Abstract

Mosquitoes (Diptera: Culicidae) pose a significant threat to public health worldwide, especially in tropical and subtropical regions, where they act as primary vectors in transmission of infectious agents. In Peru, 182 culicid species have been identified and several species of the genus *Culex* are known to transmit arboviruses. However, knowledge of mosquito diversity and distribution remains limited, with many studies focusing on specific regions only. Here, we describe a new morphological variation of *Cx.* (*Culex*) *coronator* Dyar and Knab, 1906, and report the presence of *Culex* (*Carrollia*) *bonnei* Dyar, 1921 in the central region of Peru, Huanuco. Specimens were obtained through larvae collections and identified through morphologic characterization, including dissection of male genitalia, and molecular analyses. In total, 17 mosquitoes were analyzed, and the genitalia of the male specimens allowed the identification of *Cx. coronator* and *Cx*. *bonnei*. Partial sequences of the *CoxI* gene corresponding to these two species were obtained (*N* = 10). Phylogenetic analysis revealed that the sequences of *Cx. coronator* grouped in a monophyletic clade with sequences ascribed to other species corresponding to the subgenus *Carrollia*, while *Cx. bonnei* specimens formed a monophyletic clade with homologous sequences from GenBank. This study underscores the importance of continued efforts to study the diversity and distribution of mosquitoes in Peru, including their potential role as vectors of human pathogens, to underpin effective disease control and prevention strategies, highlighting the importance of a complemented morphological and molecular analysis.

**Supplementary information:**

The online version contains supplementary material available at 10.1007/s13744-024-01160-7.

## Introduction

Mosquitoes of the Culicidae family represent a threat to public global health, especially in tropical and subtropical regions, where they participate as the main vectors in the transmission of infectious agents that affect humans and animals. Of the circa 3700 species of mosquitoes worldwide, 1069 species are registered in the neotropical region (Wilkerson et al. 2021), of which in Peru, 182 species have been officially reported; of these, 44 belong to the genus *Culex* Linnaeus, 1758 (Ayala et al. 2020, 2021) and many of these are considered arbovirus vectors, especially the species of the subgenus *Melanoconion* (Turell et al. 2005; Yanoviak et al. 2005; Turell et al. 2006, 2008; Evangelista et al. 2013; Hang et al. 2016; Treangen et al. 2016; Turell et al. 2021). In Peru, the arboviruses transmitted by *Culex* spp. mosquitoes include Mayaro virus (MAYV) (Andreolla et al. 2022), Venezuelan equine encephalitis virus (VEEV) (Aguilar et al. 2007; Vilcarromero et al. 2010), Oropouche virus (OROV) (Silva et al. 2019; Martins et al. 2020), Peruvian horse sickness virus (PHSV), Yunnan virus (YUOV) (Méndez et al. 2015), and Guaroa virus (GROV) (Aguilar et al. 2010).

Furthermore, to correctly access the risk of vector borne diseases (VBD), and carry out its surveillance and control, it is important to know the potential vector species that are present. Therefore, it is necessary to both update the registry of the mosquito species in addition and characterize their range of distribution and ecology (Pagac et al. 2021). Morphological analysis of male mosquito genitalia allows accurate identification of most mosquito species (Yadav et al. 2014; Sallum et al. 2020), as they are less susceptible to damage. In addition, the morphological analysis of the genitalia of male mosquito specimens helps to differentiate specimens that are members of species groups whose female specimens are indistinguishable, and even for which, sometimes, genetic analyses of some markers fail to disclose species identity unambiguously (Shaikevich and Vinogradova 2014).

Otherwise, with the development of molecular biology techniques, the study of mosquito DNA has become an important tool for the resolution of many taxonomic discussions (Bejarano 2001). One of the molecular tools used in the identification of mosquitoes is via DNA barcoding (Hebert et al. 2003a), the most widely used being the 658 bp partial sequence of the mitochondrial *cytochrome c oxidase I* subunit (*CoxI*) gene (Hebert et al. 2003b).

The department of Huanuco is located in the central-eastern region of Peru, covering an area of 37,266 km^2^, which represents 2.9% of the national territory. This department has two natural regions: the *sierra* (mountain) with 22,150 km^2^ and the mountain jungle, with 15,116 km^2^, and an altitude which can range between 160 and 3850 m above sea level.

The climate in this department according to the Köppen-Geiger classification is “Af” (tropical climate with forest rain) (Peel et al. 2007). In this department, 36 species of mosquitoes have been reported (Ayala et al. 2020). Of these, six species correspond to the genus *Culex* (*Culex archegus* Dyar, 1929; *Cx*. *corniger* Theobald, 1903; *Cx*. *declarator* Dyar and Knab, 1906; *Cx*. *habilitator* Dyar and Knab, 1906; *Cx*. *mollis* Dyar and Knab, 1906 and *Cx*. *urichii* Coquillett, 1906). However, the species record has had few updates and most of these have resulted from studies which were carried out more than four decades ago (Morales 1971), creating a large gap in our current knowledge of the culicine distribution in the region.

This work reports a new morphological variation of *Cx. coronator*, and the first record of *Cx. bonnei* in the mountainous region of the department of Huanuco, Peru.

## Methods

### Mosquito sampling

**Fig. 1:**
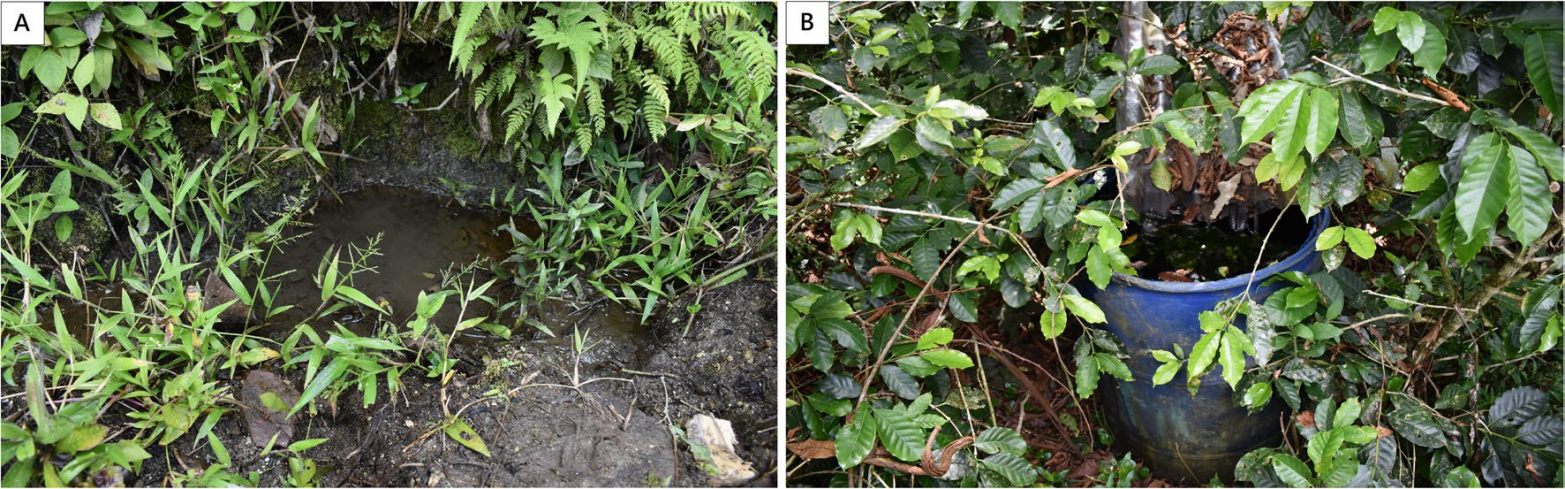
Photograph of mosquito larvae breeding places: **A** artificial container with abundant organic matter, **B** natural water deposit on the ground formed by a puddle

**Fig. 2:**
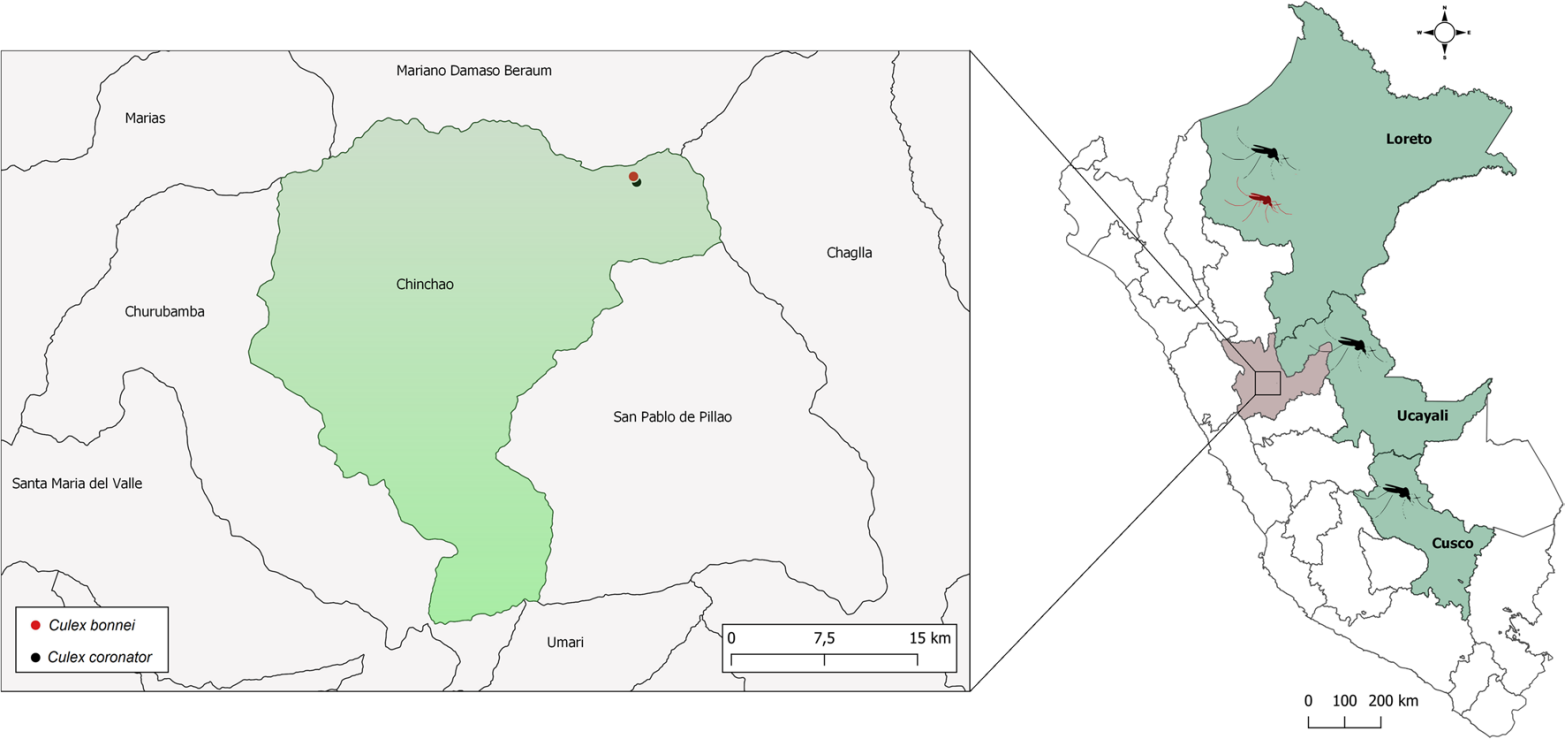
Map of Peru, as depicted in the right panel, illustrating the administrative departments where specimens of *Culex coronator* and *Culex bonnei* (depicted by black and red silhouettes, respectively) were previously collected. Within the Huanuco department, as shown in the left panel, the approximate locations where *Culex coronator* and *Culex bonnei* were collected are denoted by red and black dots, respectively, specifically within the Chinchao district

On October 7, 2022, 20 mosquito larvae were collected in two breeding places. The first was an artificial container with abundant organic matter (leaves), approximately 92 cm in height and 60 cm in circumference that was used to collect rainwater for the cultivation of coffee (9′30″31.08″S/75′58″51.12″W) (Fig. 1A), in which five larvae were collected. The other was a natural water deposit on the ground formed by a puddle (9′30″46.00″S/75′58″42.95″W) (Fig. 1B), in which 15 larvae were collected, the two collection points belonged to the village of Expedición, district of Chinchao, province and department of Huanuco (Fig. 2). The Expedición village is located within the accessible high jungle ecozone in the central region of Peru where plots of coffee cultivation can be found.

The collected specimens were placed in 200-ml flasks, with water from these habitats, labelled and transported to the Simulid Laboratory of the Hermilio Valdizan National University, Huanuco. Larvae were fed with fish food and kept at room temperature with 12 h of natural light. Up on emergence of adults, these were preserved in Eppendorf tubes with silica gel. Adult specimens were identified to genus and subgenus level with the help of dichotomic keys (Lane 1953; Bram 1967; Valencia 1973). For species identification, the genitalia of the male specimens were dissected and mounted on a slide with Neo-Mount fixation medium (anhydrous mounting medium for microscopy) and observed under a Leica DM1000LED microscope with a Leica MC190HD digital camera and were analyzed following the same identification keys.

### Molecular analysis

Total genomic DNA was extracted from 10 adult whole mosquitoes, following the methodology of Collins et al. (1987). For the partial amplification of the *CoxI* gene, the specific primers LCO1490 and HCO2198 were used, with the PCR conditions as described by Folmer et al. (1994). The amplified PCR products were purified and sequenced by the Sanger method (STABVida, Lda. 2825-182 Caparica, Portugal). The sequences obtained were edited with the Chromas tool version 2.6.6 (https://technelysium.com.au/wp/, accessed on January 20, 2023).

The analysis of *CoxI* sequences included the search for their homologues in the public genomic databases (GenBank/ENA/DDBJ) performed with the BLASTn tool (https://blast.ncbi.nlm.nih. gov/Blast.cgi, accessed January 20, 2023) and the taxonomy search engine in the BOLDSystems v4 database (https://www.boldsystems.org/index.php/IDS_OpenIdEngine, accessed January 20 of 2023). *CoxI* sequence analysis also included the reconstruction of their evolutionary relationships by phylogenetic inference. The latter included the construction of multiple sequence alignments using the G-INS-i iterative refinement method implemented in MAFFT v7 (Katoh et al. 2019). The obtained alignments were treated with Gblocks (http://phylogeny.lirmm.fr/phylo_cgi/one_task.cgi?task_type=gblocks, accessed January 20, 2023) after selecting the most permissive editing options.

Phylogenetic analyses were carried out using two different approaches: the maximum likelihood (ML) optimization criterion and a Bayesian framework. For both approaches, the choice of the best nucleotide substitution model (GTR + Γ: GTR-General Time Reversal; Γ-Gamma distribution) was carried out using the MEGA X software (Kumar et al. 2018). For the ML phylogenetic reconstruction, the IQ-tree software (Nguyen et al. 2015) was used and the topological support of the branches in the obtained phylogenetic trees was assessed using bootstrap analysis and an approximate likelihood ratio test [aLRT], as implemented in IQ-tree. In both cases, 1000 replicates of the original sequence data were used, and bootstrap or aLRT values ≥ 75 (% of total number of replicates) were used as indicative of strong topological support.

For the Bayesian phylogenetic inference analysis, the BEAST v1.10.4 software (Suchard et al. 2018) was used. This analysis consisted of two independent Markov Monte Carlo chains (MCMC) that were run until 1 × 10^8^ states were reached with sampling occurring at each 10,000 MCMC step (10% of which were later discarded as burn-in). Chain convergence was assessed using Tracer v1.7.1 software (http://beast.bio.ed.ac.uk/tracer, accessed 23 January 2023), which was also used to verify an adequate effective sample size (ESS) greater than 200 (after removal of burn-in). The distribution of the tree was summarized using the TreeAnnotator v1.8.3 software as a maximum clade credibility (MCC) tree, using median heights as the heights of the nodes in the tree. All phylogenetic trees were visualized using the FigTree v1.4.2 software (http://tree.bio.ed.ac.uk/software/figtree/, accessed 23 January 2023). Posterior probability values ≥ 0.80 were considered to indicate strong topological support. In both trees, the *CoxI* sequence of the species *Aedes aegypti* (Linnaeus, 1762) (MK265729.1) was used as outgroup.

The analysis of the average intraspecific and interspecific genetic divergence was calculated using genetic distances corrected with the Kimura 2-parameter (K2P) model, as implemented in the MEGA X software.

## Results and discussion

**Fig. 3:**
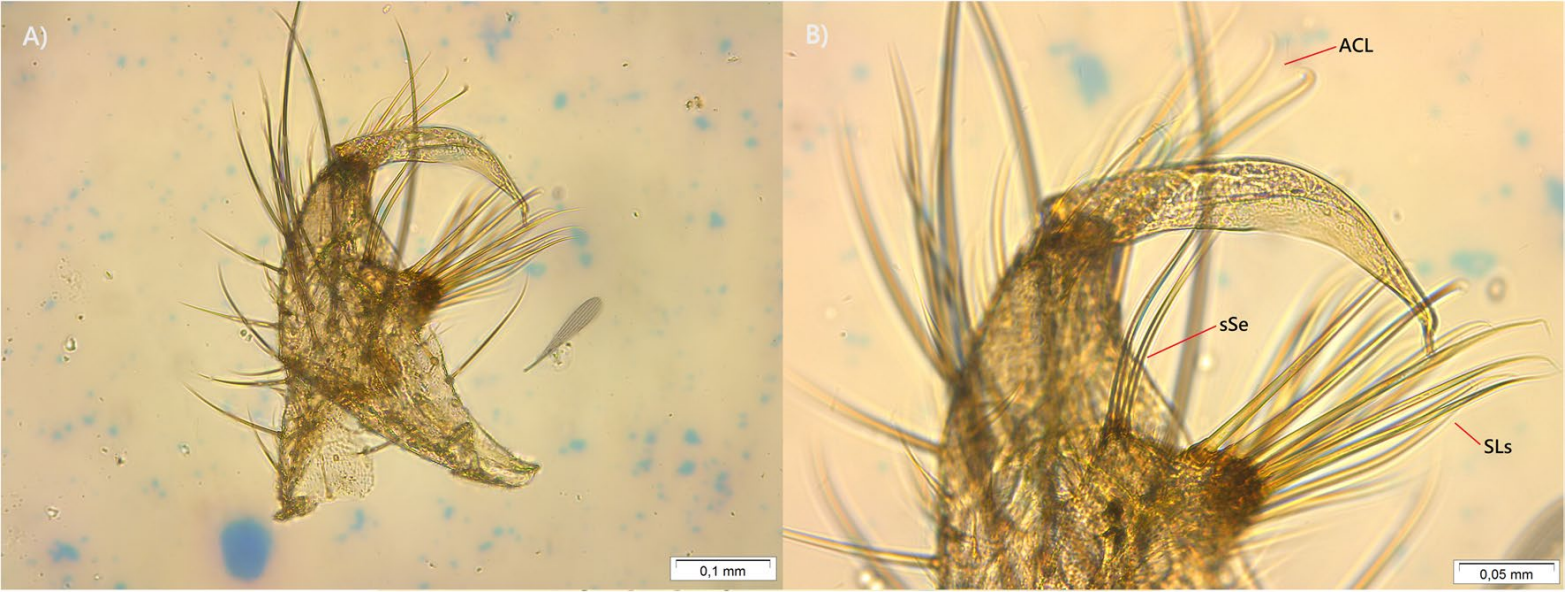
**A**, **B** Male mosquito genitalia *Culex* (*Culex*) *coronator* Dyar and Knab, 1906 with 100 × and 200 × magnification, sSe, single separated seta; ACL, apical cluster of setae; SLs, subapical lobe setae

**Fig. 4:**
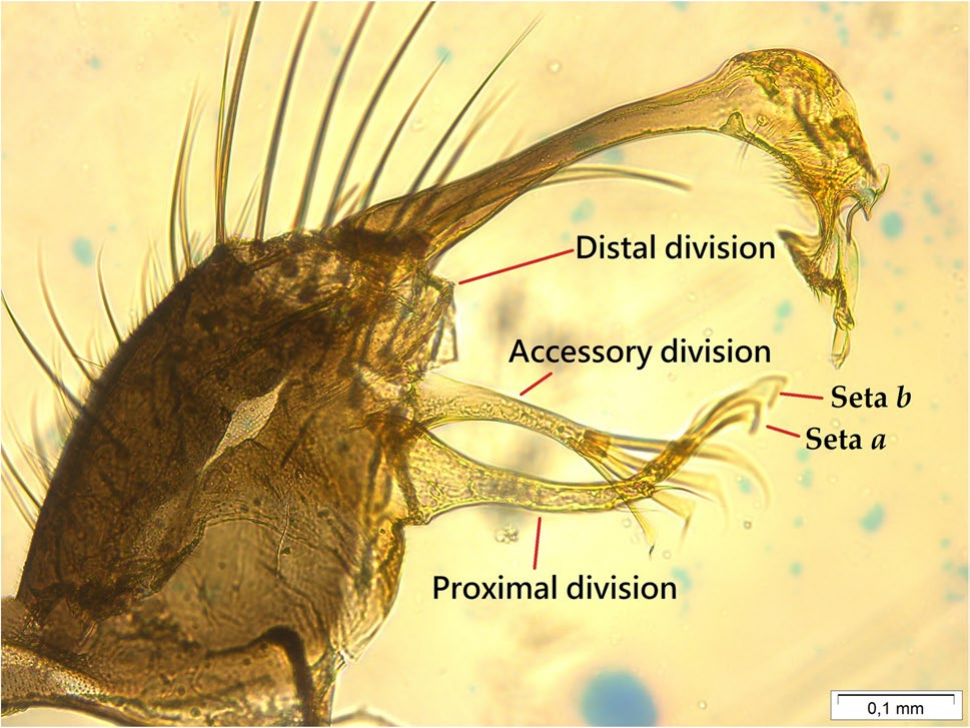
Male mosquito genitalia *Culex* (*Carrollia*) *bonnei* Dyar, 1921 with 100 × magnification; the red lines indicate the structures: distal division, accessory division, proximal division, and seta *a* and *b*

A total of 17 adult mosquitoes emerged from collected larvae; of these, 12 were identified as *Cx.* (*Culex*) *coronator* Dyar and Knab, 1906 (five males and seven females) and five as *Cx*. (*Carrollia*) *bonnei* Dyar, 1921 (three males and two females), based on morphological features and confirmed with the analysis of the genitalia of the male specimens (Figs. 3 and 4). For our study, only male specimens of *Cx. coronator* and all specimens of *Cx. bonnei* (Table 1). The slide with the mounted genitalia was deposited within the collection of the medical entomology laboratory of the National University Hermilio Valdizan, Huanuco, Peru.

**Table 1:** Data from date of collection, mosquito species, habitat, sex, collection coordinates, sequence accession number, and number of nucleotides per sequence of mosquitoes collected in Peru

Location	Collection code	Data of collection	Habitat	Species	Sex	Coordinates	nt	Access numbers
Peru, Huanuco, Chinchao	EM2-a1	October 7, 2022	Puddle	*Culex* (*Culex*) *coronator*	Male	9°30′46,00″S/75°58′42,95″W	631	LC750482
	EM2-a2	October 7, 2022	Puddle	*Culex* (*Culex*) *coronator*	Male	9°30′46,00″S/75°58′42,95″W	633	LC750483
	EM2-a3	October 7, 2022	Puddle	*Culex* (*Culex*) *coronator*	Male	9°30′46,00″S/75°58′42,95″W	616	LC750484
	EM2-a4	October 7, 2022	Puddle	*Culex* (*Culex*) *coronator*	Male	9°30′46,00″S/75°58′42,95″W	634	LC750485
	EM2-a5	November 7, 2022	Puddle	*Culex* (*Culex*) *coronator*	Male	9°30′46,00″S/75°58′42,95″W	633	LC750486
	EM4-a1	October 7, 2022	Artificial container	*Culex* (*Carrollia*) *bonnei*	Male	9°30′31,06″S/75°58′51,12″W	634	LC750487
	EM4-a2	October 7, 2022	Artificial container	*Culex* (*Carrollia*) *bonnei*	Male	9°30′31,06″S/75°58′51,12″W	633	LC750488
	EM4-a3	October 7, 2022	Artificial container	*Culex* (*Carrollia*) *bonnei*	Male	9°30′31,06″S/75°58′51,12″W	634	LC750489
	EM4-a4	October 7, 2022	Artificial container	*Culex* (*Carrollia*) *bonnei*	Female	9°30′31,06″S/75°58′51,12″W	633	LC750490
	EM4-a5	October 7, 2022	Artificial container	*Culex* (*Carrollia*) *bonnei*	Female	9°30′31,06″S/75°58′51,12″W	634	LC750491

*Culex coronator* was previously considered part of the Coronator complex, along with *Cx. camposi* Dyar 1925, *Cx. ousqua* Dyar, 1918, *Cx. usquatissimus* Dyar, 1922, and *Cx. usquatus* Dyar, 1922 (Harbach 2011; Wilkerson et al. 2021). However, Laurito et al. (2018), after dissecting the genitalia of lectotype male specimens of *Cx. coronator* and examining slides of holotype males of *Cx. camposi* and *Cx. ousqua*, as well as the male lectotype of *Cx. usquatissimus* and *Cx. usquatus*, reported a great variation, and together with the molecular evidence led them to conclude that *Cx. coronator* is a single polymorphic species with no support for the specific status of those five nominal forms (Laurito et al. 2018), supporting earlier findings by Dyar (1925).

*Culex coronator* has a wide distribution, extending from Argentina to the United States (Wilkerson et al. 2021), and can be found over a large range of breeding sites: stagnant or slow-moving water in ground pools and seeps, ditches, culverts, artificial containers, ground depressions, tire ruts, and even dredge sites, most commonly in open, sunlit aquatic habitats (Schluep et al. 2023). In this study, *Cx. coronator* larvae were collected in a ground puddle, with no other mosquito larvae present. The dissected genitalia of the specimen’s male reported in this study showed different features than those described by Laurito et al. (2018). The subapical lobe of the gonocoxite was observed without division, in addition to three robust setae on the distal margin, with 11 finer setae distributed evenly, with similar length and a small curvature at the distal end (SLs, Fig. 3B). Likewise, there is a small protuberance distal to the subapical lobe with two relatively robust setae (sSe, Fig. 3B). On the other hand, long setae are observed at the apex of the gonocoxite, with a length greater than half of the gonostylus (Fig. 3A, B). These features are very similar to those described by Bram (1967), in specimens which they considered *Cx. camposi* (Fig. 12 – c, pg. 52), although Laurito et al. (2018) found errors in this identification for not taking into consideration the holotype of *Cx. camposi*. In view of the conclusions by Laurito et al. (2018), we have but to consider that the specimens here described, found in Peru, are a form of *Cx. coronator*, compatible with the morphological variations of this species already reported by other authors (Demari et al. 2014; Laurito et al. 2018).

*Culex coronator* was implicated as a potential vector of the West Nile Virus (WNV), Saint Louis Encephalitis Virus (SLEV), Venezuelan Equine Encephalitis Virus (VEEV), Murutucu Virus (MURV), and Itaqui Virus (ITQV), in Peru, Brazil, and the United States (Consoli and Oliveira 1994; Alto et al. 2014; Demari et al. 2015; Turell et al. 2021). In Peru, *Cx. coronator* was reported in the eastern region (Loreto, Cusco, and Ucayali), although some of these reports were attributed as *Cx. usquatissimus*, *Cx. usquatus*, and *Cx. camposi* (Morales 1971; Ayala et al. 2020 and Bram 1967). However, to date, no *CoxI* gene sequences have been reported for this species in Peru.

**Fig. 5:**
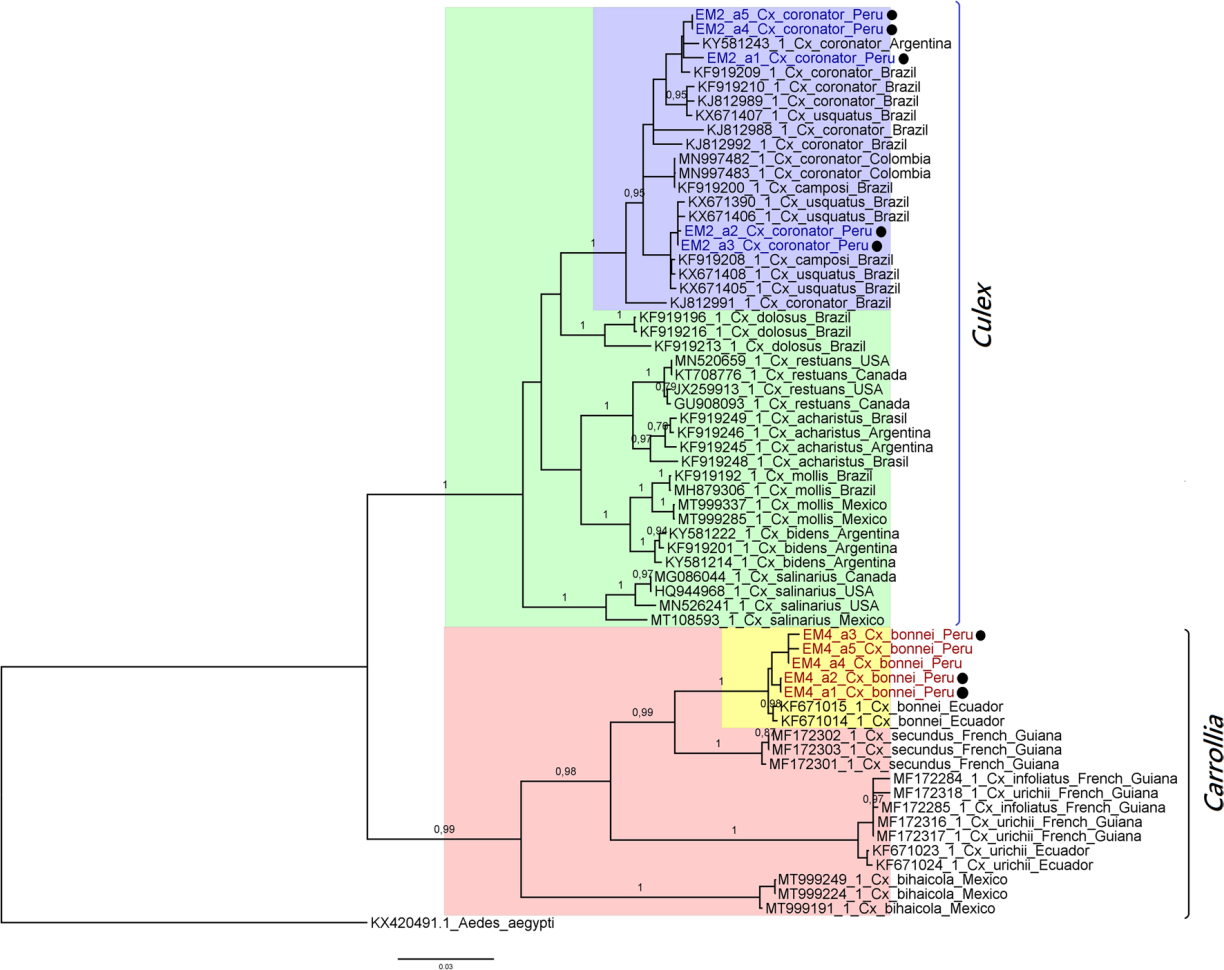
Phylogenetic analysis by Bayesian inference under the GTR + G model; the analysis involved 64 nucleotide sequences. Support values correspond to a posteriori probability. The size bar indicates 0.03 replacements per site. The sequences obtained in this work have been designated with the “EM” code, in red and blue, while the black circle indicates that they are sequences corresponding to male mosquitoes, associated with genitalia assembly. Reference sequences downloaded from the public databases and are shown by their respective access number, as well as country of origin. Vertical lines mark the *Culex* and *Carrollia* subgenera

**Fig. 6:**
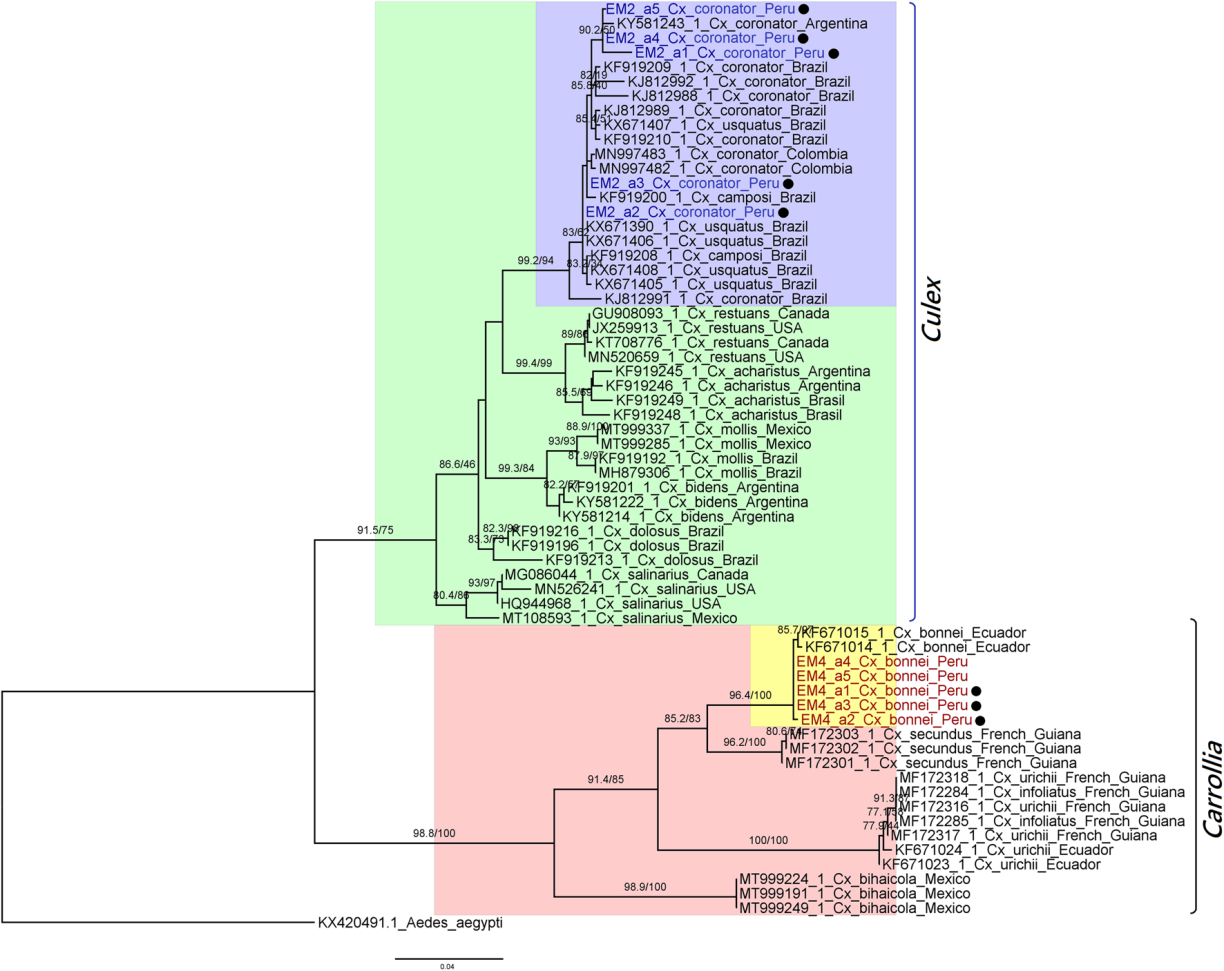
Phylogenetic analysis with maximum likelihood tree under the GTR + G model; the analysis involved 64 partial *CoxI* nucleotide sequences from *Culex*; consensus tree probability was − 3073.525. Support values for the branches were estimated with aLRT/Bootstrap with 1000 repetitions for each method. The size bar indicates 0.04 replacements per site. The sequences obtained in this work have been designated with the “EM” code, in red and blue, while the black circle indicates that they are sequences corresponding to male mosquitoes, associated with genitalia assembly. Reference sequences downloaded from the public databases and are shown by their respective access number, as well as country of origin. Vertical lines mark the *Culex* and *Carrollia* subgenera

Partial sequences of the *CoxI* gene of all five male specimens *Cx. coronator* (LC750482-LC750486) were obtained. By using the BLASTn tool, these sequences showed 99.84–100% identity with homologous sequences from *Cx. usquatissimus*, *Cx. usquatus*, and *Cx. coronator*. When using the Boldsystems taxonomy tool, an identity of 99.36 to 100% with the homologues from *Cx. usquatissimus*, *Cx. usquatus*, *Cx. coronator*, and *Cx. maxi* Dyar, 1928 was observed. Regarding the phylogenetic reconstruction analysis, the generated trees showed that our *Cx. coronator* was grouped with GenBank sequences attributed as *Cx. coronator*, *Cx. usquatus*, and *Cx. camposi* (Figs. 5 and 6); in addition, the divergence analysis between these sequences is rather low (0.79 ± 0.21 to 0.72 ± 0.18) (Table S1-1) coinciding with what has already been reported (Demari et al. 2015, 2017; Laurito et al. 2013, 2018; Vesgueiro et al. 2011).

*Culex bonnei*, of the subgenus *Carrollia* Lutz, 1904, is so far considered a mosquito species without medical importance. The subgenus *Carrollia* is divided into two groups: the Bihaicolus Group composed of five species (*Cx. bihaicolus* Dyar & Núñez Tovar 1928; *Cx. guerreroi* Cova García, Sutil, & Pulido 1971; *Cx. infoliatus* Bonne-Wepster & Bonne 1920; *Cx. metempsytus* Dyar 1921 and *Cx. rausseoi* Cova García, Sutil O, & Pulido F. 1972) and the Iridescens Group composed of thirteen species, which in turn is divided into two subgroups: Iridescens Subgroup with eleven species (*Cx. antunesi* Lane & Whitman 1943; *Cx. babahoyensis* Levi-Castillo 1953; *Cx. bonnei* Dyar 1921; *Cx. cerqueirai* Valencia 1973; *Cx. insigniforceps* Clastrier & Claustre 1978; *Cx. iridescens* (Lutz 1905); *Cx. kompi* Valencia 1973; *Cx. secundus* Bonne-Wepster & Bonne 1920; *Cx. soperi* Antunes & Lane 1937; *Cx. wannonii* Cova García & Sutil O. 1976 and *Cx. wilsoni* Lane & Whitman 1943) and Urichii Subgroup with two species (*Cx. anduzei* Cerqueira & Lane 1944 and *Cx. urichii* (Coquillett 1906)) (Valencia 1973; Wilkerson et al. 2021). In our study, we followed on the characteristics of the mosquito male genitalia, in which the subapical lobe was observed, in agreement with that described by Valencia (1973), with three divisions: the distal division formed by a small bump, the accessory division in the form of a robust and long column that ends with four long and flattened setae, and the elongated and slightly curved proximal division with two to three simple distal setae and seta *a* and *b* with dilated and curved apex (Fig. 4). In Peru, five species of *Culex* of the *Carrollia* subgenus were reported, including *Cx. bonnei*, *Cx. urichii*, *Cx. infoliatus*, *Cx. iridescens*, and *Cx. bihaicolus* (Ayala et al. 2020, 2021). So far, *Cx. bonnei* has only been only reported in northern countries of South America (Wilkerson et al. 2021) reaching the 3rd parallel south in the region northeastern from Peru (Iquitos) (Lopes et al. 1985; Pecor et al. 2000). In this study, we reported for the first time *Cx. bonnei* in the central region of Peru (Huanuco) to the 9th parallel south. This species has been reported in many types of breeding: Broken or cut bamboo, tree holes, fallen palm spathes, fallen cacao pod, fallen fruit, artificial containers metal and plastic (Valencia 1973; Lopes et al. 1985; Patrick et al. 2002). In this study, the larvae were collected in an artificial container of plastic; furthermore, we did not find it associated with other species.

Five partial *CoxI* gene sequences were obtained corresponding to the specimens identified as *Cx. bonnei* (3 males and 2 females) (LC750487–LC750491), and these showed 90.53–99.84% identity with homologues from *Cx. bonnei* in BLASTn tool, and an identity of 99.35 to 99.84% with *CoxI* sequences from this same species when the Boldsystems tool was used, thus, differently from the previous specimens, confirming its taxonomic identity. In the phylogenetic analysis, these sequences were clearly clustered with the *Cx. bonnei* from Ecuador used as reference, forming a monophyletic group strongly supported, in a clade grouping the subgenus *Carrollia*, sister to the subgenus *Culex* clade (Figs. 5 and 6). Regarding the divergence analysis, the results showed an intraspecific variation of 0.13 ± 0.09, a lower result to that obtained by Demari et al. (2011) who reported a value of 0.6, while interspecific divergence value that varied between 4.83 ± 0.92 and 10.23 ± 1.44 with *Cx. bihaicola*, *Cx. infoliatus*, *Cx. urichi*, and *Cx secundus* sequences (Table S1-1). On the other hand, it is observed that the grouping of the sequences does not correspond to the proposed informal groups. In other studies, only one or two species of this subgenera were included in their phylogenetic analysis, which limited the observation of this grouping (Demari et al. 2011; Linton et al. 2013; Viveros et al. 2022).

Studies regarding the analysis of mosquitoes from Peru are limited, outdated, and concentrated only in some regions of the country, which generates a gap in the knowledge of the distribution, ecology, and diversity of the species over the Peruvian national territory (Ayala et al. 2020). In the department of Huanuco, 15 genera including 36 species of Culicidae have been registered, and of these, six are *Culex* species. Our study reports a new form of *Cx. coronator*, in addition to reporting for the first time *Cx. bonnei* in this region in Peru, besides contributing with partial *CoxI* gene sequences of these species originating from Peru to GenBank, highlighting the importance of using both morphological and molecular tools in mosquito identification (Montalvo et al. 2022, Mixão et al. 2016). However, we suspect an underestimation of mosquito biodiversity, since no further studies on culicids have been carried out in this region since 1971, even though they are important vectors of human and/or animal pathogens and whose presence is affected by the activities of humans (Johnson et al. 2008; Gorris et al. 2021).

## Supplementary information

Below is the link to the electronic supplementary material.

Supplementary file1 (DOCX 62 KB)

## Data Availability

The slides with the mounted dissected genitalia of the mosquitoes in this study are deposited in the Faculty of Veterinary Medicine and Zootechnics of the Hermilio Valdizan National University.
