# Supplementary material for: Description of New Morphological Variation of Culex (Culex) coronator Dyar and Knab, 1906 and First Report of Culex (Carrollia) bonnei Dyar, 1921 Found in the Central Region of Peru
Source: Neotrop Entomol. 2024 Jun 25;53(4):987–96. doi: 10.1007/s13744-024-01160-7 (PMC11255002; doi:10.1007/s13744-024-01160-7)
Supplement: Supplementary file 1 — Supplementary file1 (DOCX 62 KB) [file 13744_2024_1160_MOESM1_ESM.docx]

1. Intraspecific and interspecific variation based on the analysis of partial *coxI* sequences amplified. The indicated values have been calculated using the K2P model.

1. Intraspecific and interspecific variation based on the analysis of partial *coxI* sequences of some species Coronator complex from different countries. The indicated values have been calculated using the K2P model.

|  | | Nº of sequences | Intraspecific variation (%) | | Interspecific variation (%) | | | | | | | | | |
| --- | --- | --- | --- | --- | --- | --- | --- | --- | --- | --- | --- | --- | --- | --- |
|  |  |  |  |  | 1 | | 2 | | 3 | | 4 | | 5 | |
| 1 | *Cx. camposi* Brazil | 2 | 0,49 | ± 0,27 |  |  |  |  |  |  |  |  |  |  |
| 2 | *Cx. usquatus* Brazil | 5 | 0,29 | ± 0,15 | 0,34 | ± 0,15 |  |  |  |  |  |  |  |  |
| 3 | *Cx. coronator* Peru | 5 | 0,53 | ± 0,18 | 0,69 | ± 0,22 | 0,64 | ± 0,21 |  |  |  |  |  |  |
| 4 | *Cx. coronator* Colombia | 2 | 0,16 | ± 0,16 | 0,49 | ± 0,23 | 0,43 | ± 0,21 | 0,5 | ± 0,18 |  |  |  |  |
| 5 | *Cx. coronator* Brazil | 6 | 1,39 | ± 0,29 | 1,01 | ± 0,24 | 0,91 | ± 0,20 | 1,1 | ± 0,22 | 1,02 | ± 0,26 |  |  |
| 6 | *Cx. coronator* Argentina | 1 | n/c | n/c | 0,65 | ± 0,28 | 0,74 | ± 0,32 | 0,68 | ± 0,26 | 0,72 | ± 0,32 | 1,16 | ± 0,30 |

1. Intraspecific and interspecific variation based on the analysis of partial *coxI* sequences of subgenus *Carrollia* from different countries. The indicated values have been calculated using the K2P model.

|  | | Nº of sequences | Intraspecific variation (%) | | Interspecific variation (%) | | | | | | | | | | | |
| --- | --- | --- | --- | --- | --- | --- | --- | --- | --- | --- | --- | --- | --- | --- | --- | --- |
|  |  |  |  |  | 1 | | 2 | | 3 | | 4 | | 5 | | 6 | |
| 1 | *Cx. bihaicola* Mexico | 3 | 0,00 | ± 0,00 |  |  |  |  |  |  |  |  |  |  |  |  |
| 2 | *Cx. urichi* French Guiana | 3 | 0,22 | ± 0,15 | 12,69 | ± 1,62 |  |  |  |  |  |  |  |  |  |  |
| 3 | *Cx. infoliatus* French Guiana | 2 | 0,00 | ± 0,00 | 12,56 | ± 1,61 | 0,11 | ± 0,08 |  |  |  |  |  |  |  |  |
| 4 | *Cx. urichi* Ecuador | 2 | 0,32 | ± 0,24 | 12,86 | ± 1,65 | 0,54 | ± 0,26 | 0,65 | ± 0,32 |  |  |  |  |  |  |
| 5 | *Cx. secundus* French Guiana | 3 | 0,11 | ± 0,11 | 9,48 | ± 1,38 | 8,95 | ± 1,27 | 8,82 | ± 1,27 | 9,11 | ± 1,30 |  |  |  |  |
| 6 | *Cx. bonnei* Ecuador | 2 | 0,16 | ± 0,16 | 10,42 | ± 1,47 | 9,58 | ± 1,37 | 9,58 | ± 1,38 | 9,19 | ± 1,35 | 5,13 | ± 0,98 |  |  |
| 7 | *Cx. bonnei* Peru | 5 | 0,00 | ± 0,00 | 10,12 | ± 1,45 | 9,48 | ± 1,36 | 9,48 | ± 1,37 | 9,09 | ± 1,34 | 4,86 | ± 0,95 | 0,24 | ± 0,19 |
